# Methodology for quantitative rock characterisation using multiple imaging systems and random particles generation

**DOI:** 10.1016/j.mex.2022.101807

**Published:** 2022-08-03

**Authors:** Pia Lois-Morales, Catherine Evans, Dion Weatherley

**Affiliations:** a The University of Chile, Faculty of Physical and Mathematical Sciences, Department of Mining Engineering, Santiago, Chile; b The University of Queensland, Sustainable Minerals Institute, WH Bryan Mining and Geology Research Centre, Australia; c The University of Queensland, Sustainable Minerals Institute, Julius Kruttschnitt Mineral Research Centre, Australia

**Keywords:** Texture, mineralogy, Ore characterisation, Image processing

## Abstract

The characterisation technologies have advanced rapidly in the last decade. From a qualitative observation of minerals with optical microscopy, more quantitative techniques have emerged. Examples are the SEM-based technologies that focus on mineralogical identification at the microscale and the X-ray microtomography systems that allow identifying rock features in three dimensions.

Features such as rock texture and mineralogy have a degree of control on how the rock behaves in the processing plant and thus can affect the project's economic feasibility. None of the available measurement devices is currently capable of identifying all the aspects of rock characteristics that are of interest in linking mineralogy and texture to process response in a single measurement. However, through the integrated use of the techniques in a complementary approach is possible to generate the required suite of information about the mineralogical composition and mineral grain size and shape in a given sample. A multisource method for rock characterisation has been developed in this work. This method includes:

• A multistage imaging process that uses 2D and 3D microscopes

• An object-segmentation technique to separate mineral grains in the photomicrographs for the quantification of mineralogical and textural properties.

• A segmentation technique was developed to create particles of different sizes from a larger image.

## Graphical abstract

**Figure:**
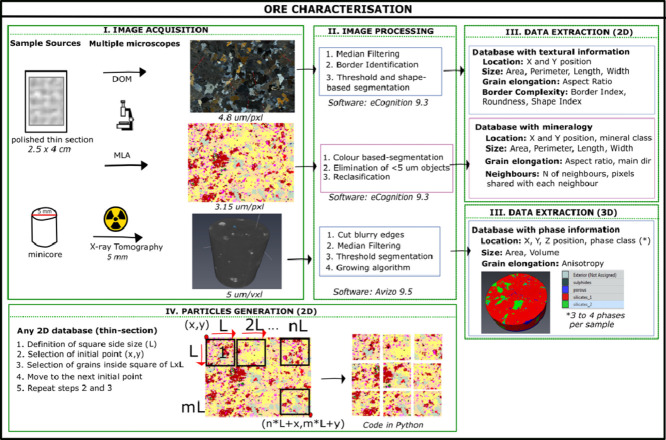

**Specifications table**

**Table:** 

Subject area;	
More specific subject area;	*Image analysis*
Name of your method;	*Quantitative characterisation of rock particles generated by a random segmentation method with no replacement*
Name and reference of original method;	*N.A.*
Resource availability;	*Software eCognition: http://www.ecognition.com/*

## Method conceptualization

Recent decades have observed a rapid advance in the technologies for capturing high-resolution information from rocks at different scales. Advanced characterisation techniques, such as automated mineralogy systems based on scanning electron microscopes, have specialised in the mineralogical identification of thin-polished rock sections [10,11]. Other techniques, such as the X-ray micro-tomography systems, allow the characterisation of particles in three dimensions, removing the stereological bias present for decades in the imaging technologies [14], [15], [16]. Long-standing techniques such as optical microscopy have been automated, and now image analysis techniques can be applied to photomicrographs to obtain quantitative measurements of features, such as grain boundaries and minerals' shape [1,12]. These techniques are dedicated to obtaining data from small size samples such as thin-polished sections or briquettes with a diameter of a few centimetres but each of them specialized in a particular piece of information.

The first section of the method presented herein focuses on using microscope techniques in a complementary way, allowing the data integration of ore characteristics from various sources. These characteristics include different measurements of grain size (e.g., length, width, area), shape (aspect ratio), relationships between grains (percentage of border associations, border complexity) and mineralogical proportion. The methodology developed combines information from different microscope systems such as the SEM-based automated mineralogy system, Mineral Liberation Analyser (MLA), Digital Optical Microscope (DOM) and an X-ray microtomography system. An image analysis procedure was used to extract quantitative information from the samples' images and create databases from each of them.

On the other hand, the process response of rocks during their treatment in mining processing plants depends on the ore characteristics, such as mineralogy, porosity, and their distribution in rocks [3,7]. However, most techniques applied to ores to understand their processing performance rely on physical testing, which, particularly in comminution, can require hundreds of kilos of material and barely consider the geological variability. The abundant information captured by the characterisation devices presents an opportunity for the processing plant to inform and predict more accurately the incoming ore variability and its effects.

The second section of this methodology considers an algorithm to simulate particles of different sizes, considering a random breakage [8] of the original photomicrographs from thin-polished sections (25×40 mm). This allows simulating a breakage process inside a comminution unit while carrying quantitative information of the ore throughout different particle sizes.

The development of other technologies that focus on quantifying mineralogy with great precision on larger samples, such as the recently developed M4 Tornado (Bruker, [30]) which measures mineralogy by processing the X-ray fluorescence signal to identify and quantify mineralogy in diamond drill core samples, or core logging characterisation technologies such as Corescan®, which integrate reflectance spectroscopy with a spectral resolution of 4 nm, high-resolution images (60 µm/pxl), and 3D laser profiling [6], is a pathway for the application of this methodology on larger scale techniques that might provide an understanding of the breakage process that undergoes in larger rock particles.

## Characterisation devices

Three different microscopy techniques (DOM, MLA, and X-ray microtomography) were used to capture different pieces of information for characterising the material. The DOM version Leica DM6000 was used for this work; its internal control system allows creating of a mosaic of photomicrographs of a thin section or a polished block. These photomicrographs are taken by a high precision stage, allowing these individual frames to be tiled precisely. The DOM also allows setting up an automatic measurement run to collect images under different imaging modes creating layers (crossed or parallel Nicols in transmitted or reflected light) (See Figure 2). The optical mineralogy has the advantage that the resultant image is an outcome of the interaction of the crystallographic orientation of the grain and the light passing through it [13]. Two grains of the same composition might have a different crystallographic orientation resulting in a different observation of their optical properties [33]. For this reason, an optical microscope is a convenient tool for identifying grain boundaries between minerals with similar chemistry, which can be used to quantify the grain size and the real shape of grains. However, the automatic identification of minerals using optical microscope images is not straightforward due to all the possible chemical differences translated into different optical properties of a mineral grain [13].

**Figure 1:**
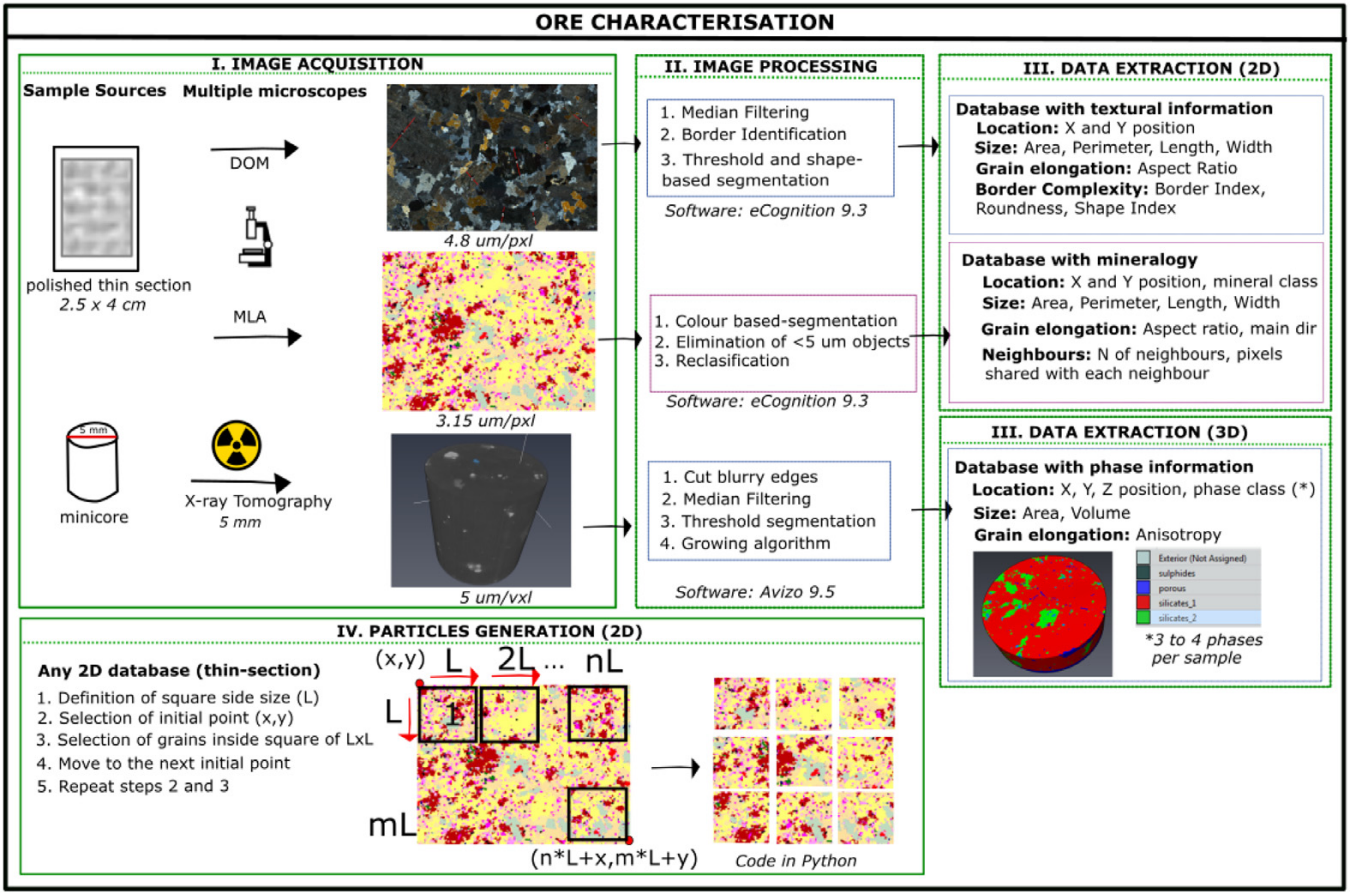
Multisource ore characterisation procedure

**Figure 2:**
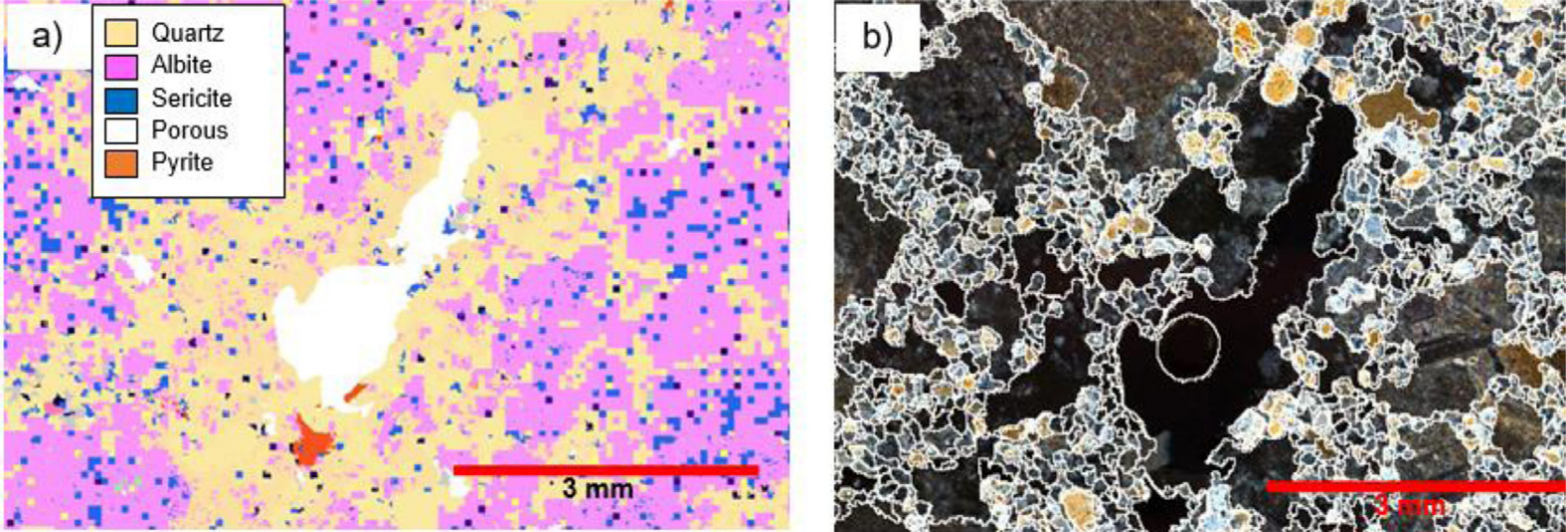
Identical regions from a thin-polished rock section measured in two systems. (a) SEM-based system. (b) DOM Image with mineral grain boundaries highlighted, modified from Lois-Morales et al. [18]

The SEM-based systems such as the MLA are commonly used for rock characterisation in the mining industry as they deliver accurate identification and quantification of major minerals. The MLA was developed at the JKMRC during the 1990s by Dr Ying Gu to quantify mineral liberation [11] and overcome the mineral identification issue. This system consists of a scanning electron microscope (SEM) fitted with energy dispersive spectrometers (EDS). So, the MLA uses backscattered electrons (BSE) signals and X-ray spectra to identify different minerals that compose the samples. This microscope is equipped with a software package that controls the SEM, capturing images and matching each mineral EDS signal to a library of X-ray spectra. The BSE grayscale signal allows differentiation of particles from the resin epoxy mounting medium and the MLA software segments the mineral grains to accelerate the identification of the minerals. One of the measurement modes incorporated in the system is the GX-MAP mode, which collects the X-rays in a grid mapping mode to discriminate minerals. It is time-consuming, but the resultant mineral map calculates precise modal mineralogy and mineral association in regions where complex intergrowths are present. The MLA allows the detection of areas or phases with detailed mineralogy. However, it cannot detect borders between grains of the same elemental composition, which can be resolved using it in combination with DOM images. Figure 2 shows a rock segment imaged with an MLA and a DOM.

**Figure 3:**
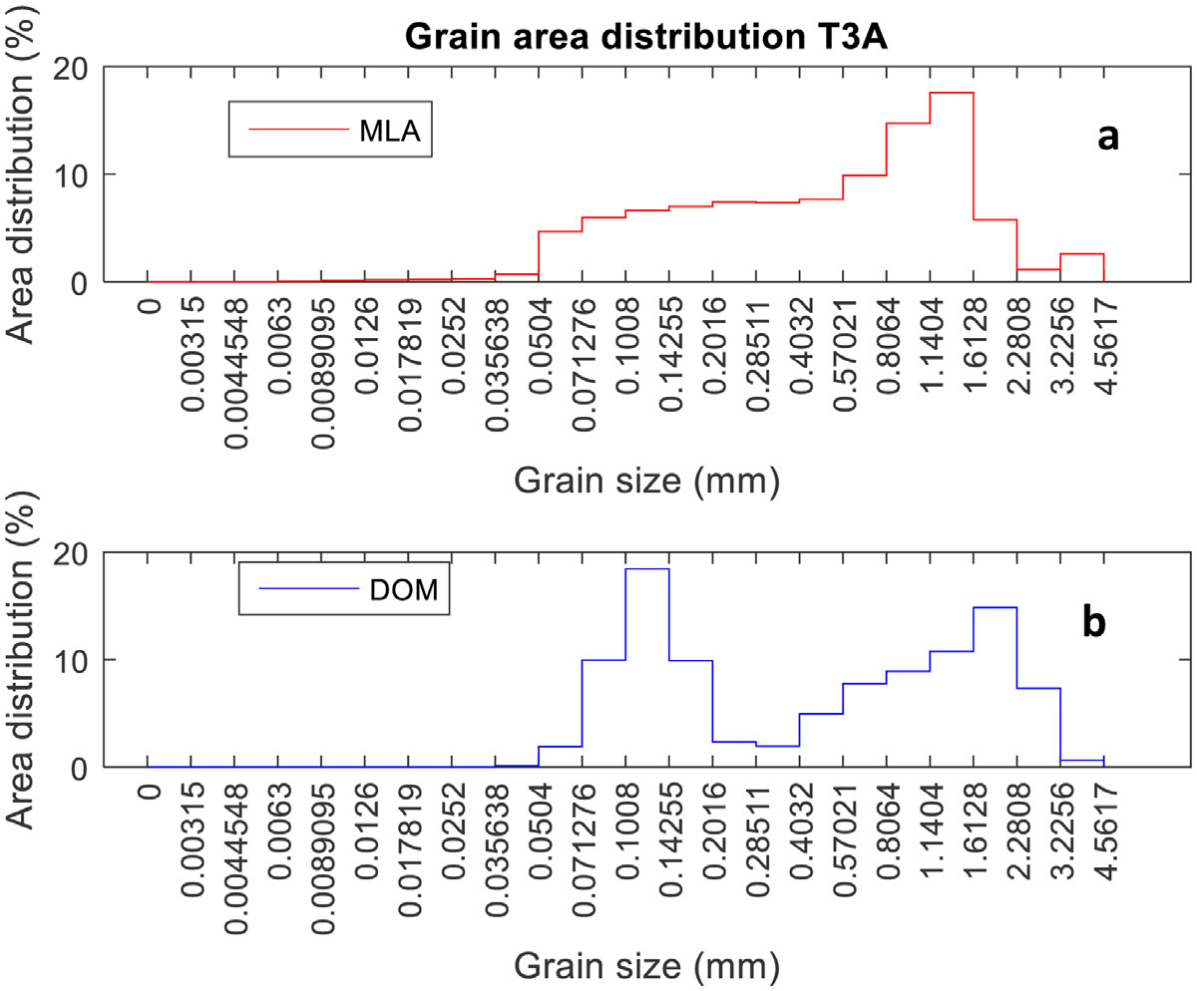
Grain Size quantification using two methods. (a) SEM-based method. (b) DOM.

The SEM image in Figure 2a shows the yellow areas, identified as quartz, as a continuous area. However, the optical microscope image in Figure 2b shows that this area is an aggregate of small quartz grains. The use of the DOM in conjunction with the MLA may allow the determination of detailed mineralogy with real grain size. The data graphed in Figure 3 shows the grain size calculated from both systems, where the DOM quantifies a bimodal distribution of grain size in Figure 3b, while a unimodal distribution is observed in the MLA data in Figure 3a.

**Figure 4:**
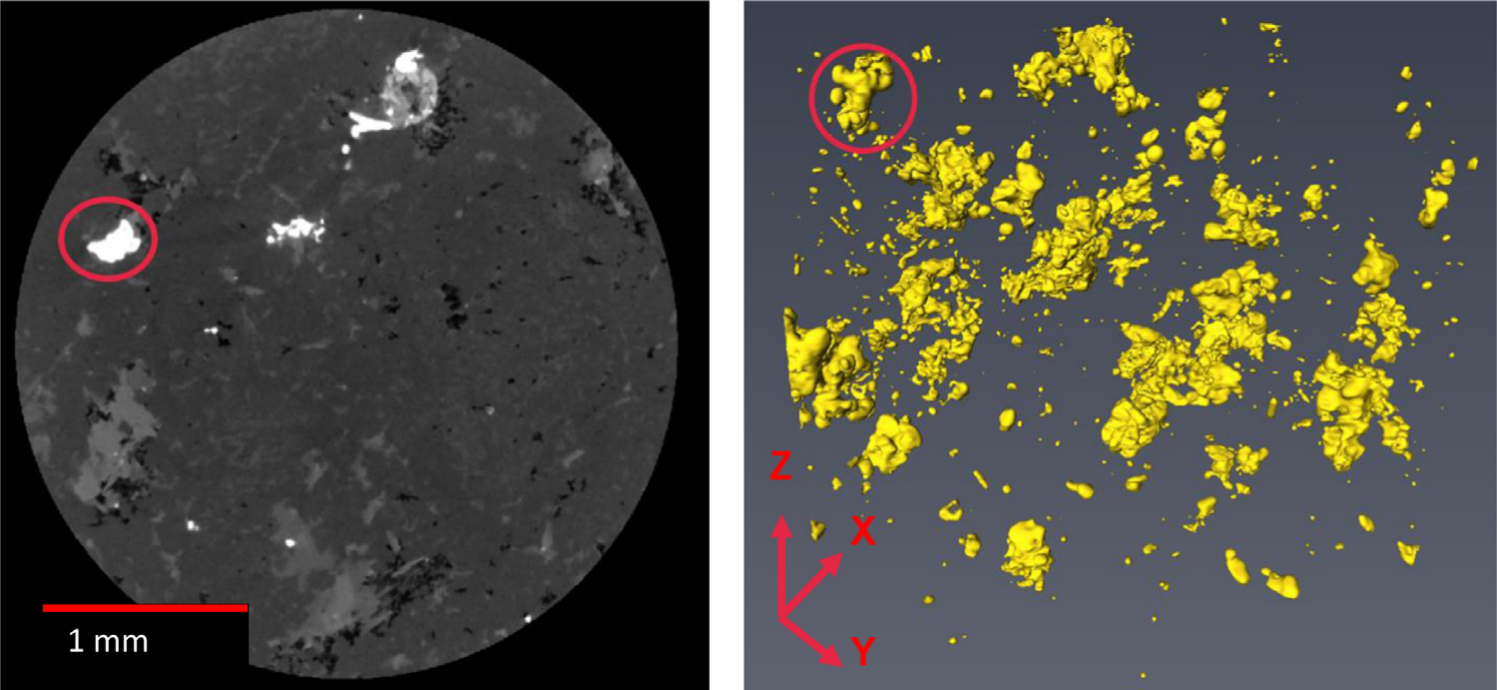
Left. X-Y 2D slice from a 3D measured Sample. Right. 3D projection of sulphides grains

Most previous studies comparing processing characteristics with quantitative geological properties have relied upon 2D images (Batchelor et al., 2015, Batchelor et al., 2016, Djordjevic, 2013. However, the stereological bias for some characteristics such as grain size is an issue associated with 2D measurements. The 3D-size of an object cannot be quantified with a 2D single projection. There are infinite possibilities where a plane can intersect the 3D volume resulting in different 2D size measurements. The example in Figure 4 shows that the size of the highlighted grain is different if it is projected in the X-Y or Y-Z direction.

The utilisation of 3D methodologies such as the X-ray microtomography system is a step forward for minerals characterisation. During the measurement time in an X-ray tomography system, the X-rays pass through the sample, and a reconstruction software uses the transmitted X-rays to create a 3D image (tomogram) of the sample, representing a series of stacked 2D image slices. In this work, the tomogram is and the 2D images have a voxel/pixel resolution of 5 µm. The most significant advantage of 3D tomography over 2D techniques is the capability to quantify the spatial location and orientation of mineral phases in three dimensions [9,15]. However, identifying minerals with similar densities, i.e. similar X-ray attenuations, is still an issue to solve [9].

**Figure 5:**
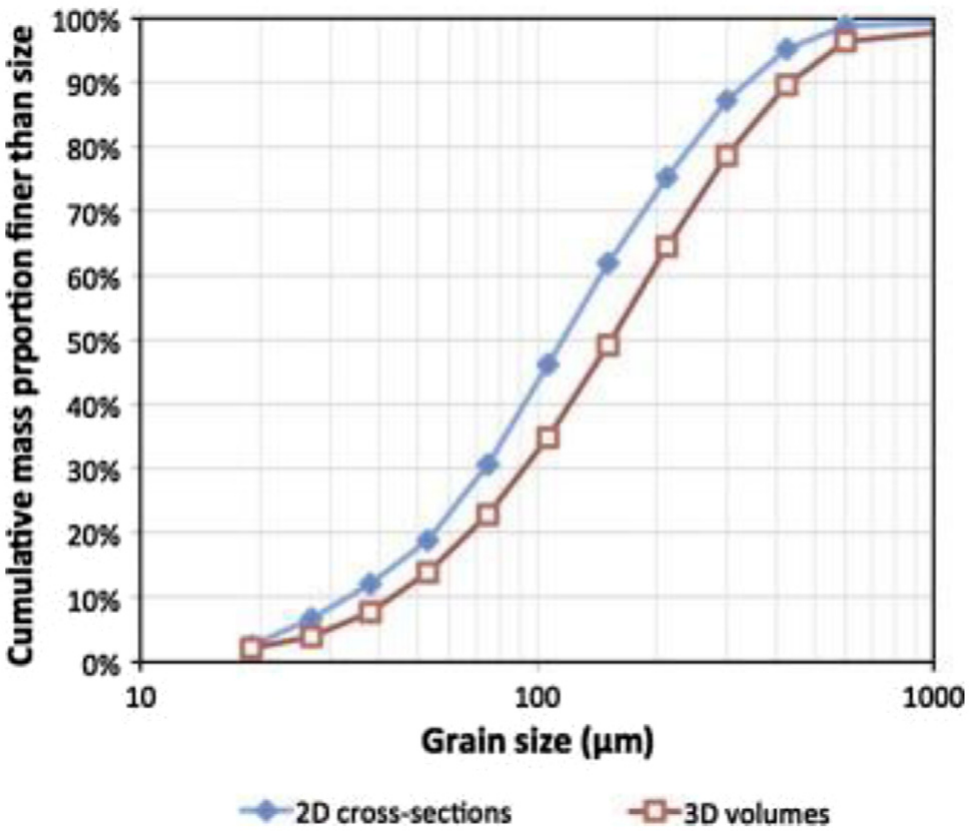
Comparison of pyrite grain size distribution from 2D and 3D image measurements. Taken from Evans et al. [9].

In the processing context, the utilisation of 3D techniques can even change the baseline conditions for designing a grinding circuit [9,28]. An example is in Figure 5, where the grain size distribution obtained from a 2D image differs from the values obtained using a 3D tomogram. This could significantly impact the P80 selected as the comminution target to liberate the valuable minerals from the gangue minerals.

**Figure 6:**
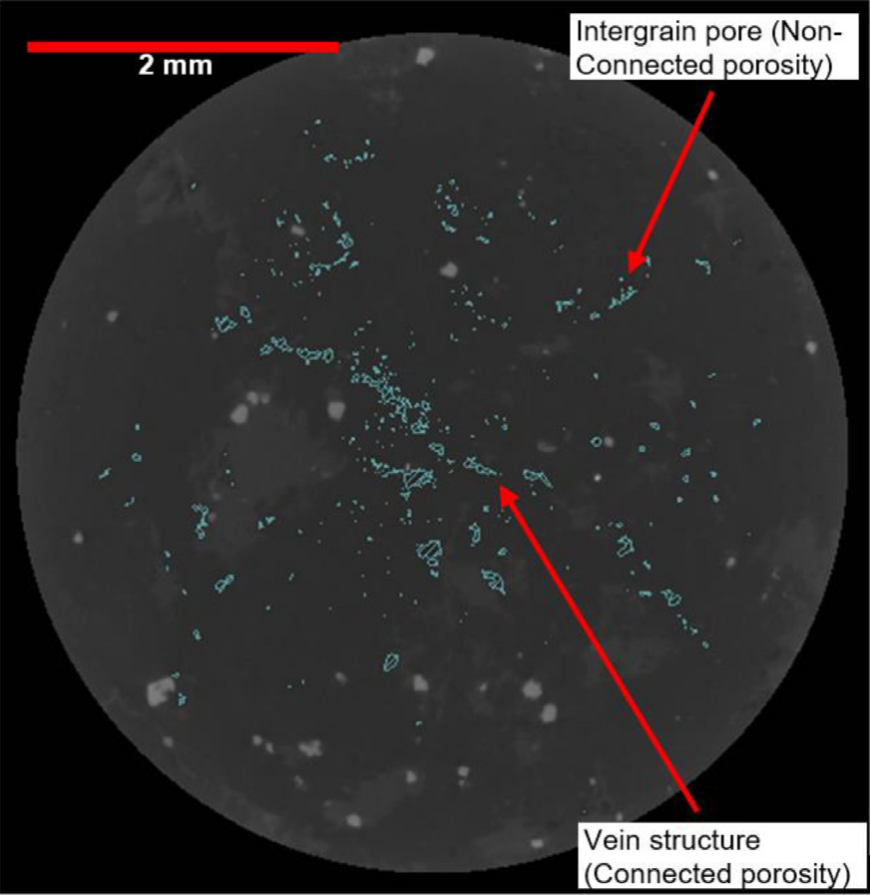
X-Ray micro-tomography 2D slice with voids highlighted.

Other researchers have also demonstrated that this methodology is beneficial for identifying and quantifying internal voids in samples [35,42]. Voids connected to the sample's surface can be easily identified with water or gas displacement techniques such as the He-Pycnometer. However, these techniques do not allow the measurement of internal pores which are not connected to the surface. By utilising the X-ray tomography system, non-connected voids can be easily quantified because the air filling the voids has a significant density difference with the rock (see Figure 6).

## Method description

After selecting rock samples, slabs of rock measuring 4×2.5×1 cm were cut for the fabrication of thin-polished sections that allow the 2D imaging process to be carried on using the Digital Optical Microscope and the Mineral Liberation Analyser. On the other hand, minicores of 5 mm were required for the 3D imaging using the X-ray tomography system. These minicores were prepared by drilling rock slabs larger than 7 cm with an SPD-25A Pedestal drill with a fixed drilling speed of 2410 rpm, using diamond drill bits of different diameters.

Four stages were applied to the thin-polished sections and the drilled minicores to transform images into particles' quantitative information, which can be separated into two main tasks. The first task considers the first three stages for transforming the information into quantitative data. First, an image acquisition process is carried on by taking photomicrographs of the samples using the different systems described above. The second stage is implemented by applying image improvement techniques such as filtering and cleaning that allow the identification of grain boundaries in the images obtained with the DOM, different mineral phases in the case of MLA and pores in the X-ray tomograms. After the image processing stage, a third data extraction stage involves adding the different objects (mineral grains, pores) that compose a rock into a structured database. There are several software packages available for performing image analysis; some examples are eCognition® (Trimble), Leica Application Suite (LAS) (Leica-Microsystems) for 2D image analysis and Avizo for 3D images (ThermoFisher). Some open-source packages that include 3D image analysis are ImageJ (NIH), Quant3D, Blob3D [16], Pore3D [4], RockCreate [17]. Also, programming languages such as Matlab and R contain image analysis tool packages that allow segmenting objects with different characteristics. In this study, eCognition® was used for analysing 2D images and Avizo for working with 3D images.

The second task of the method and the fourth stage of the data extraction process is used for the simulation of the segmentation of the fracture process by segmenting the information extracted from the thin-section databases into smaller databases that represent smaller pieces of the thin-polished sections or particles. This second task has only been applied to the information extracted from the 2D images but will be applied to 3D data in future work. This last stage of segmentation is carried out using Python code, which can be modified depending on the size of the available images and the size of the particles required for the study.

### Part 1: Extraction of quantitative information

#### Stage 1: Image acquisition

**Figure 7:**
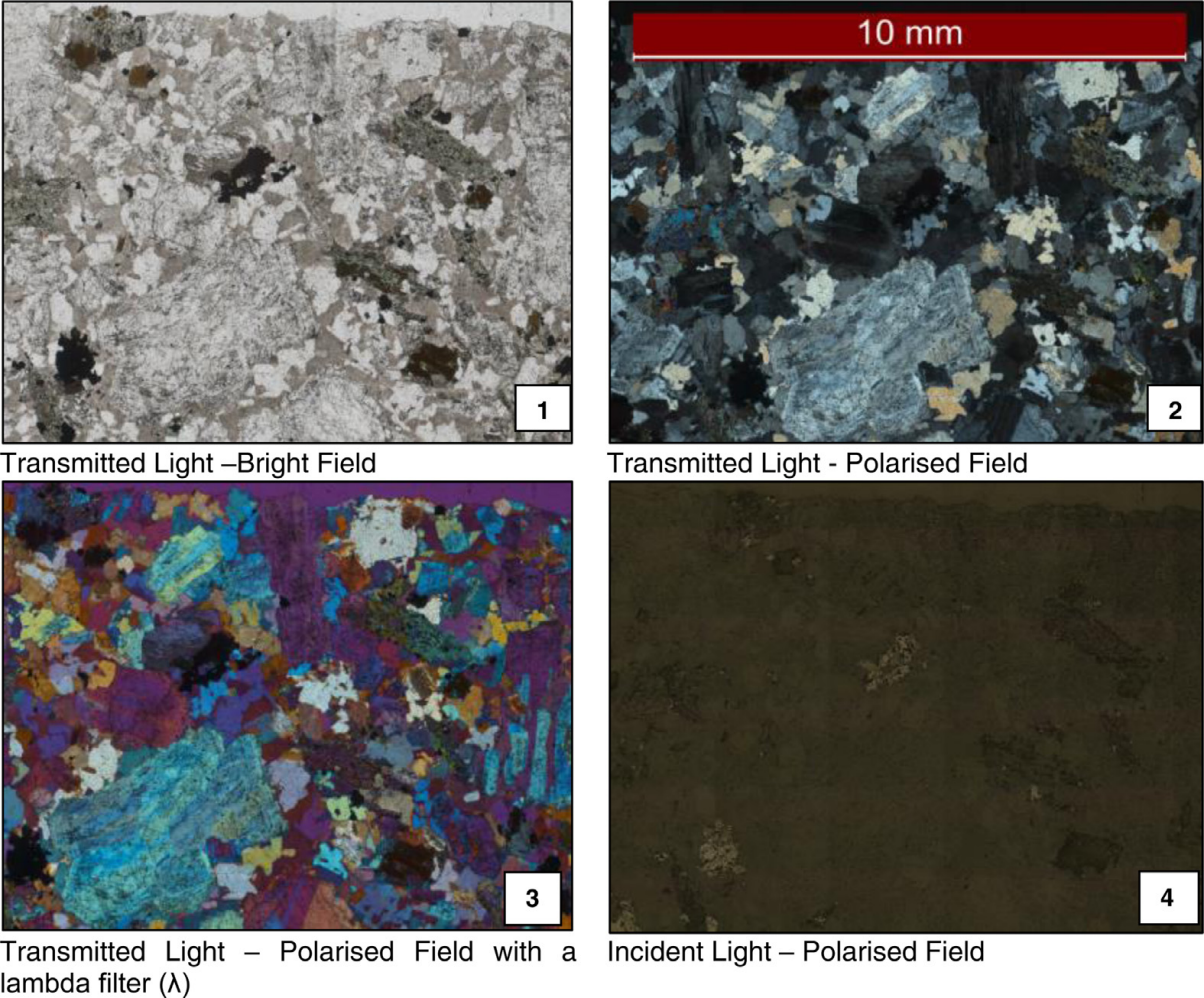
Example images of the four imaging modes of the digital optical microscope

The thin-polished sections are imaged using an optical microscope model Leica DM 6000, controlled by the Leica Application Suite software (LAS v.12). Each thin-polished section is scanned using a mosaic mode available in the software, which enables the microscope to scan the thin section by controlling the movement of the stage and using a grid to collect multiple photomicrographs. Approximately 300 photomicrographs are taken from one thin-polished section, which is then tiled into one single image. The image's resolution obtained with the DOM depends on the lens magnification; the 5x magnification used in this study allowed a resolution of 4.8 µm per pixel. All illumination conditions are kept constant during the imaging process, such as condenser aperture and exposure settings avoiding any saturated pixels. All thin-polished sections were imaged with four different microscopy light modes (See Figure 7) for creating a four-layer registered image as suggested by Hartner [12], Bonnici [2] and Berry et al. [1] in the P843 – GEM^III^ AMIRA project [5].

The same thin-polished sections measured in the DOM system are carbon-coated and analysed in the MLA using the GX-MAP mode with a magnification of 150X and a step size of 16×16 pixels; the resultant images have a resolution of 3.15 µm/pixel. Images from the electron microscope are processed and classified using the following colour scheme (See Table 1).

**Table 1:** RGB values of colour codification of MLA images

ID	Mineral	R	G	B	ID	Mineral	R	G	B
1	Ag sulphides	90	90	90	13	phosphates	0	0	128
2	background	255	255	255	14	plagioclase	255	255	117
3	carbonates	0	97	0	15	pores	255	251	240
4	chalcopyrite	128	0	0	16	Py - Aspy	166	202	240
5	others	128	0	128	17	pyrosmalite	255	168	255
6	fluorite	235	235	235	18	quartz	252	234	160
7	gypsum	158	158	207	19	talc	255	0	255
8	inosilicates	189	0	0	20	clays-ser-talc	255	0	255
9	K Felsdpar	253	225	159	21	sulphates	87	192	255
10	micas	192	220	192	22	anorthite	255	192	255
11	other sulph	0	128	0	23	albite	255	150	255
12	oxides	255	0	0	24	other Cu sulph	128	0	0
					25	orthoclase	253	225	159

In addition, the 5 mm diameter cylindrical minicores are imaged using an X-ray microtomography system model Zeiss Xradia Versa 500. X-ray measurement conditions (i.e., input energy and collecting X-ray time) were set differently for each sample, in order that e the image quality as the density of the minerals generate different coefficients of attenuation of X-rays can be optimised [14], [15], [16]. The resultant greyscale tomogram is a function of this attenuation coefficient, where minerals with similar densities display similar greyscale values. This slight difference in greyscale makes it difficult the discrimination between minerals with similar densities, but the identification of voids from the rock matrix is easier as voids do not attenuate the x-rays, so they are easily distinguishable from rock material.

#### Stages 2 and 3: Image processing and data extraction

Once the images from thin-polished sections and minicores have been collected with the different microscopes, these are transformed into numeric databases using an image analysis routine.

##### Image processing and data extraction from Digital Optical Microscope images

The four registered photomicrographs obtained from each thin-polished section (See Figure 7) are processed using the eCognition® software. The extraction of quantitative data from the optical microscope's images is challenging as the optical properties of minerals can easily change with a minimal change in their chemistry. Therefore, this procedure focuses on identifying the mineral borders instead of generating a mineralogical type of characterisation. Identifying mineral borders allows the quantification of grain size, shape and border complexity of mineral grains.

**Figure 8:**
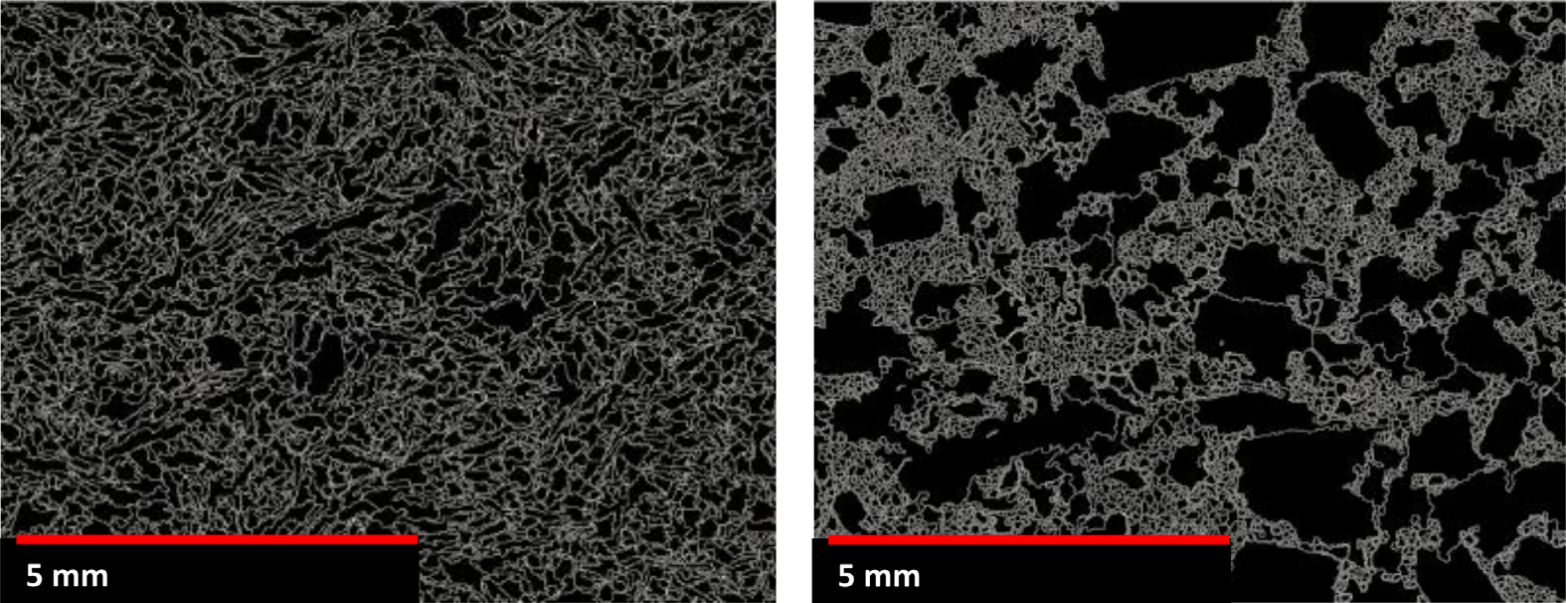
Left: Mineral grain boundaries image from S1-A. Right: Mineral grain boundaries image from S3-B.

Each image is loaded in eCognition® as a combination of layers of RGB values; therefore, an image of a thin section has twelve bands of information. A ruleset for segmenting the multilayer images based on the code of Berry and his team for the P843 – GEM^III^ AMIRA project [1,5] was developed in eCognition®. Firstly, a median filter with a neighbour of 3 pixels is applied to the images to remove their noise. Then, a contrast filter is used over the images for enhancing the borders between minerals. An object-segmentation process is applied over the filtered images, segmenting the objects representing each mineral or void that composes the rock. This segmentation process is carried out using a multi-resolution algorithm in several steps. The algorithm uses homogeneity as a criterion for separating areas, which are controlled by a predetermined size. The number of segmentation steps is dependent on the variability of the grain size. If a large initial size is defined, e.g., coarse grain size, the segmentation process will look for homogeneity across a larger number of pixels. So, several steps considering different initial sizes were carried out in sequence for rocks with variable grain sizes. The weight of the layers during the segmentation process can also change depending on the targeted mineral. A segmentation where the incident light photomicrograph (See Figure 7D) has more weight than the other layers allows better segmentation of metallic minerals. An example of the resultant segmentation process is observed in Figure 8, where the white lines represent the boundaries between different grains of rocks.

Figure 8 shows two studied textures, S1 on the left has a fine unimodal grain size (∼1 mm) and S3 on the right has a bimodal grain population with coarse grains (>5 mm) and a family of grains under 1 mm. In these figures, each grain is individually characterised. Once the borders between grains are demarked, each grain is identified with a position in the X-Y space and is characterised by different indices. Table A1 in Annexe summarises the most common indices for quantifying ore characteristics using two-dimensional (2D) images; only some of them were selected for this study.

**Figure 9:**
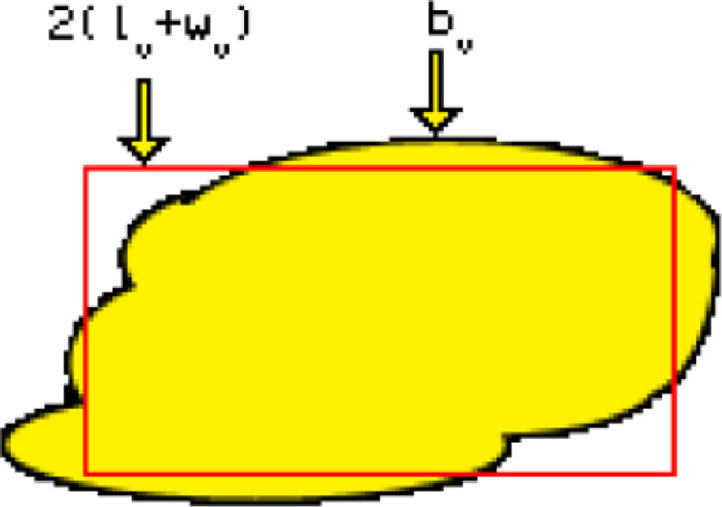
Border Index graphic representation (from Trimble [40])

• Grain Location: inner_x, inner_y, are the centre position of the grain on the x and y-axis.
• Grain class: grains are classified according to their area ((coarse >50000 pixels, >11.5 mm^2^), fine (<10000 pixels, <2.3 mm^2^) or medium (between those two area values)).
• Grain size: this variable is described by variables such as Area (A), Border Length (L), length ($D_{l}$), width ($D_{W}$). The area corresponds to the number of pixels that cover the whole grain. $D_{l}$ corresponds to the number of pixels that covers the longest axis in the grain and $D_{W}$ to the number of pixels on the shortest axis. The border length corresponds to the number of pixels forming the perimeter of the grain. These measurements can be transformed into metric units by multiplying the image's resolution (4.8 µm/pxl).
• Grain elongation: this is described by the Aspect ratio of each grain (Ar) which is calculated from the size data as the ratio between the length and the width of the grain (See Eq. 1)
  (1) $$A_{r}=\frac{D_{l}}{D_{W}.}$$
• Border complexity or tortuosity is estimated using the Border Index (BI) parameter (Eq. 2). The BI describes the irregularity of the border using a rectangular approximation (See Figure 9) and is calculated as the ratio between the border perimeter (L) and the perimeter of the smallest enclosing rectangle, where l and w are the sides of the rectangle (a value of one corresponds to a rectangular object).
  (2) $$\mathrm{BI}=\frac{L}{2}*\left(l+w\right)$$

The information extracted from each thin-polished section is exported to an individual numerical (.csv) database, where each grain identified in that thin-polished section is described by its central pixel location and the variables mentioned above.

##### Image processing and data extraction from Mineral Liberation Analyser images

The colour-coded GX-Map images generated from the MLA measurements are post-processed with the eCognition® software (Trimble). The image processing considers a classification and a segmentation stage for extracting quantitative information. The classification process assigns a mineral class to each coloured pixel, according to Table 1. Then, an object segmentation based on the RGB colour scheme is carried on for separating each phase and assigning a position in space and the descriptive indices mentioned below. Because this segmentation is based on the fixed colour scheme, the eCognition® script does not change considerably from one rock to another. In contrast with the DOM images processing, the boundaries between grains with the same composition cannot be identified in the MLA images, and only mineral phases are distinguishable. Once the segmentation of objects is performed, each of them is described with the following information:

• Phase Location: inner_x, inner_y, are the centre position of the crystal in the x and y-axis.
• Phase mineral name: correspond to the mineral class assigned by the MLA to each pixel in the image.
• Phase Size: Area (A), Border length (L).
• Grain elongation: The aspect ratio of each grain (Ar) was calculated from the size data (Length/Width), and the Form factor (Ag) or Roundness can also be calculated for each rock.
• Neighbours: number of neighbours (NN), shared a border with each neighbour (NB)
• The number of neighbours' information refers to the number of mineral phases surrounding each mineral phase on its Border length (L).
• The shared border between grains refers to the number of shared pixels between each crystal and each of its neighbour phases. This value can be transformed into a percentage by dividing the total number of pixels in the grain boundary by each grain's total border (pixels).

This information extracted from each MLA-measured thin-polished section is then extracted into individual databases (.csv), where each phase is identified with the information above.

##### Image processing and data extraction from X-ray Tomography images

The image processing stage of the 3D tomograms from the X-ray microtomography measurements is performed with the AVIZO 9.7 software (ThermoFisher). The main objective of this procedure is to identify the total proportion of voids present in the rock and to characterise their size and orientation. The segmentation and quantification of the rock characteristics required several steps. First, the tomogram is cut to select a Volume of Interest (VOI) using a volume editing tool that eliminates the diffuse sample borders. Like the optical microscope images, a median filter with a neighbour of 3 voxels is applied to the tomograph to reduce the image noise, which preserves better the object borders and size. A multi-thresholding segmentation algorithm is used to discriminate between mineral phases. A visual check of the greyscale is used to perform an initial threshold set-up for each phase. Then, the algorithm automatically searches the best segmenting range from the initial given point. Between three and four phases can be recognised for each rock type using this procedure. The application of this procedure in altered granitoid samples allowed the identification of three phases (silicates, metallic minerals, and pores). However, in the case of the basalt samples, the high content of ferromagnesian minerals allowed the separation of silicates into inosilicates/ferromagnesian micas and tectosilicates. Each segmented object contains the following quantitative information:

• Phase Identification (tag): 1) Pores, 2) Silicates, 3) Metallic Minerals as an example.
• Phase location: X, Y, Z values correspond to the coordinates of the centre position of the phase on the x-, y- and z-axis in μm.
• Phase Size: described by the area and volume of the sample.
• Area 3D (A): Correspond to the number of pixels that cover the surface area of the phase.
• Volume 3D (V): Correspond the number of voxels that represent the volume of the phase.
• Phase Elongation: Anisotropy (An) of each phase measures the deviation of the region from a spherical shape (See Eq. 3). It is calculated as one minus the ratio between the pixels in the shortest direction of the crystal versus the pixels in the longest direction of the crystal. An anisotropy value of zero corresponds to a spherical grain. This index also analyses the elongation of the crystal as the Aspect ratio (Ar) described in Eq. 2.
  (3) $$An=1-\frac{1}{A_{r}}$$

Once the phases are segmented, the 3D numerical information described for each of the imaged cores is exported into individual numerical databases in .csv file format.

### Part 2: Particles generation

The image analysis procedures described above are used to extract the information from the thin-polished sections, typically with a dimension of 4 cm by 2.5 cm. The last data processing stage and the second part of the methodology implemented an algorithm to create particles using the thin-sections information. This algorithm uses a resampling without a replacement process, representing a random breakage behaviour of the material, with no preferential direction or predominance of fractures [8,20]. This segmentation process creates smaller sections of the thin-polished image section called "simulated particles". This process aims for representing a broader range of particles that could be formed during a size reduction process.

This random sampling approach (written in Python programming language) consists in the segmentation of the thin-polished section information using a squared sliding window of a selected size. The algorithm starts by selecting the further top left grain with a coordinate (x,y). From that position, a distance L (adjustable to any size) is measured in x and y directions to create the squared window. All the grains that had their centre inside the selected area are copied into a new database to create a new simulated particle. Then, the following initial point for segmenting the next simulated particle starts in the same row (x+L,y). The segmentation process continues in the same row until it reaches the end of the thin-polished section, where the last particle of the row starts in (x, (n-1)*L+y), with n the number of particles segmented in the x-axis. When the final squared particle on the first row is segmented, the algorithm continues in (x,y+L) until it reaches the point ((m-1)*L+x, (n-1)*L+y), being m the number of particles segmented in the y-axis.

The grains in each simulated particle carry all their characteristics (i.e., grain size, border index, mineralogy), which allows the analysis of each simulated particle. Figure 1 shows the resampling process for any MLA or DOM image using a sliding window. This procedure was only used in the information of the 2D image due to the amount of available data. However, it is underway to be implemented in 3D images when more data is compiled.

## Possible improvements to the method

**Figure 10:**
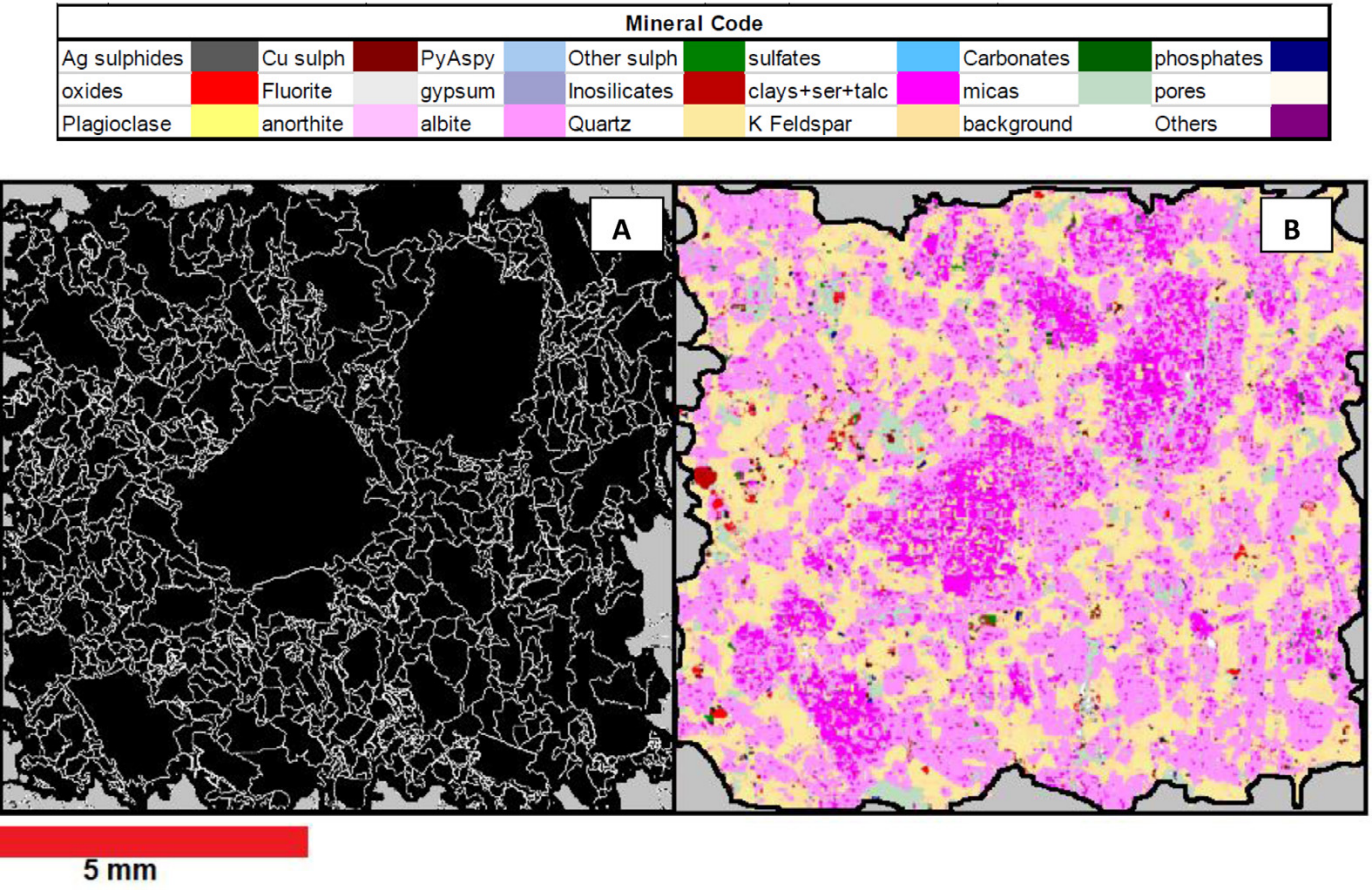
Example of a simulated particle from sample S6. A) Mineral grain borders image from Image analysis outcome from DOM microphotography. B) Classified Image from MLA

In this research, the subsampling process is applied to the numerical databases created from the 2D images instead of segmenting the images directly. Converting the images into databases before creating the simulated particles allows faster operation of the segmentation algorithm as the image processing is carried on only once. However, this procedure has a disadvantage since each grain's centre's (x,y) location is the reference point for including or discarding a grain from a sub-sampled database. The resampling process produces larger particles when the rocks have a coarser grain size. For the same reason, and because the grains have irregular borders, the shape of the particles is irregular. Mariano [19] did not observe significant differences in the liberation of ore minerals associated with the shape of the simulated particles. An example of the actual shape, where the irregularity of the boundaries can be observed, is in Figure 10.

Even though the sliding window was placed in the same initial coordinate (x,y) for MLA and DOM, the resultant segmented particles do not represent precisely the same area. Grains are segmented from the DOM images, which are usually smaller than the mineral phases segmented from the MLA. So, the objects selected when the segmentation occurs may vary slightly between the images from the different microscopes. So, each device's data is treated independently for most of the analyses. An improvement of the analysis can be made by producing particles where the same are covered with both systems.

On the other hand, random breakage might be incorrect for some samples that might break under a non-random breakage approach during breakage [20]. A further modification of the segmenting algorithm might be helpful to represent a non-random breakage condition that could be promoted by the difference in physical properties between two adjacent minerals [43].

Sampling methods with replacement or geometric masking have been applied previously to rock texture images for simulating random breakage events as described in the literature [8,19]. A sliding window procedure was used herein to create the simulated particles and ensure no overlap between them. The work was performed with five or six thin sections imaged per rock type, representing approximately 50 cm^2^ of sample in area, which might not be enough to create a sufficiently large number of particles over 10 mm side. Implementing a random sampling with replacement instead of a sliding window could also be applied to increase the number of particles, especially if more data is available.

## Conclusions

A novel characterisation methodology that combines information extracted from 2D MLA colour-coded images, photomicrographs from optical microscopy and 3D images from X-ray tomography is presented. The methodology focuses on obtaining specific, complementary information from each device which enables the importance of each mineralogical and textural characteristic over the processing behaviour of ores to be investigated. A novel segmentation method using databases instead of images has been developed as part of the work. This method simulates similar particles to those created during grinding (millimetric scale), each of which carries characterisation information. This methodology could be potentially applied to other characterisation techniques that work on other scales, with could help to represent breakage within a large range of particle sizes and provide an understanding of the rock properties affecting the size reduction process.

## Ethics statements

MethodsX has ethical guidelines that all authors must comply with. In addition, we ask you to complete the relevant statement(s) below. Please delete those which are not relevant to your work.

## Funding

This work was supported by the Chilean Agency for research and development (ANID) [grant number Becas Chile 72160275] and the Complex Ore Bodies Program of the Sustainable Minerals Institute.

## CRediT authorship contribution statement

**Pia Lois-Morales:** Conceptualization, Data curation, Writing – original draft. **Catherine Evans:** Supervision, Writing – review & editing. **Dion Weatherley:** Software, Methodology, Writing – review & editing.

## Declaration of interests

The authors declare that they have no known competing financial interests or personal relationships that could have appeared to influence the work reported in this paper.

## Data Availability

Data will be made available on request.

## Appendix Supplementary materials

Image, application 1
