# Supplementary material for: Methodology for quantitative rock characterisation using multiple imaging systems and random particles generation
Source: MethodsX. 2022 Aug 3;9:101807. doi: 10.1016/j.mex.2022.101807 (PMC9400075; doi:10.1016/j.mex.2022.101807)
Supplement: Supplementary file 1 [file mmc1.docx]

# Additional Information

Table 1: Measurements extracted from Image analysis procedures of rocks.

| Parameter | Index | Formula | References |
| --- | --- | --- | --- |
| SIZE | Perimeter (L) | Sum of all objects sides |  |
|  | Area (A) | Total Region enclosed by perimeter |  |
|  | Feret Diameter: Distance between two parallel objects that is a tangent to the object profile and perpendicular to the visual scale. Usually, 8 to 16 are calculated before to find the Feret Diameter. |  | [36] [41] [42] |
|  | Major Axis (length) $(D_{l})$: Distance between two furthest points of the mineral (x,y). | $D_{l}=\sqrt{{(x_{2}-x_{1})}^{2}-{(y_{2}-y_{1})}^{2}}$ | [43, 44] |
|  | Minor Axis (width) $(D_{w})$: Distance between two closest points of the mineral (x,y). | $D_{w}=\sqrt{{(x_{2}-x_{1})}^{2}-{(y_{2}-y_{1})}^{2}}$ | [45] [43] |
|  | Equivalent Circle Diameter ($D_{e}$): This measurement can be related to standard sieve sizes. The diameter of the circle that encloses the area calculated. | $D_{e}=\sqrt{\frac{4A}{\pi}}$ | [36, 44, 46, 47] |
|  | Particle Specific Surface Area (PSSA): Defined as the area divided by an estimated volume or Area divided by length in 2D images.*This can be performed by grain (PSSAW). | $PSSA=A/V$ | [48] (Leißner et al., 2016) [49] |
| SHAPE | Aspect Ratio $(A_{r})$: Elongation | $A_{r}=\frac{D_{l}}{D_{w}}$ | [43] [44] |
|  | Angularity (Ag) or Form Factor $(F_{f})$: its parameter that compares deviation from circularity due to elongation or roughness | $Ag=4\pi(\frac{A}{L^{2}})$ | [42] [41, 44, 50] |
|  | Roundness (Rd): Ratio of curvature of grain edges to overall grain shape. | $Rd=\frac{4A}{\pi D_{l}}$ | [43] |
|  | Sphericity (S): Comparison between Radio of circle that inscribe${(R}_{i})$ particle versus Radio of circle that circumscribes particle ${(R}_{c})$. | $S=\frac{R_{i}}{R_{c}}$ | [43] |
|  | Compactness (C): Comparison between perimeter and area of a mineral. Very similar to Ag. | $C=\frac{L^{2}}{A}$ | [43, 44] |
| ORIENTATION | Main Orientation ($\emptyset$) | The direction of the main axis of a grain | [41, 43, 44, 51] |
|  | Angle Factor Orientation $(F_{a})$: It Is calculated by a weighted class system applied to the absolute, acute angular difference $\left( {90}^{o}-\emptyset\right)$, between every elongated grain. N is total elongated particles, Xi is the number of angular differences in each class. i is the weighting factor | $F_{a}=\sum_{i=1}^{9} (\frac{x_{i}}{\frac{N(N-1)}{2}})*i$ | [41, 43, 44, 51] |
| BORDER COMPLEXITY | Degree of Interlocking $(D_{i})$: Border compared with the area. | $D_{i}=\frac{1}{n}\sum_{i=1}^{n} \frac{L_{i}}{\sqrt{A_{i}}}$ | [41, 43, 44, 51] |
|  | Grain Boundary Smoothness or Roughness (GBS): Comparison between the perimeter of an enclosed circle with the real perimeter. | $GBS= \frac{L_{\mathrm{circle}}}{L_{\mathrm{real}}}$ | [41, 43, 44, 51] |

Table 2: Measurements extracted from 3D Image analysis of rocks.

| Parameter | Index | Formula | References |
| --- | --- | --- | --- |
| SIZE | Volume *(V)* | Total Region enclosed by Area |  |
| SIZE | Area *(A)* | The surface of the object |  |
| SHAPE | Aspect Ratio (AR): Is a measurement of the shape of the particle | First of all, it is necessary to define the central moment of the particle as:  $\mu_{p,q,r}=\sum_{x} \sum_{y} \sum_{z} {(x-x_{g})}^{p}{(y-y_{g})}^{q}{(y-y_{g})}^{r}$  Where x, y, z are the coordinates of each voxel and x_g_, y_g_, z_g_ are coordinates of the centre of gravity.  Then eigenvalues of the matrix formed can be associated with the length, width and thickness of the grain.   | [35] |
| SHAPE | Sphericity (S): A measurement of the similarity of the particle to a sphere | $S= \frac{\sqrt[3]{36V^{2}}}{A}$ | [35] |
| ORIENTATION | Angle Factor Orientation $(F_{a})$: Orientation ($\emptyset$) of elongated grains. | Similar to $F_{a}$ in 2D but calculated with the longest direction in 3 dimensions | [41, 43, 44, 51] |
